# Asymmetric Adaptability to Temporal Constraints Among Coordination Patterns Differentiated at Early Stages of Learning in Juggling

**DOI:** 10.3389/fpsyg.2018.00807

**Published:** 2018-05-23

**Authors:** Kota Yamamoto, Masahiro Shinya, Kazutoshi Kudo

**Affiliations:** ^1^Department of Life Sciences, Graduate School of Arts and Sciences, The University of Tokyo, Tokyo, Japan; ^2^Graduate School of Integrated Arts and Sciences, Hiroshima University, Higashihiroshima, Japan; ^3^Graduate School of Interdisciplinary Information Studies, The University of Tokyo, Tokyo, Japan

**Keywords:** juggling, adaptability, intrinsic patterns, sensorimotor synchronization, individual differences, motor learning

## Abstract

In this study, we examined the degree of adaptability to new constraints after learning of a fundamental skill in juggling. Adaptation of sensorimotor synchronization with the various constraints is important for expertise. However, this adaptability may not be equivalent between coordination patterns which learners acquired in the previous learning process. In other words, there may be “asymmetric” adaptability among intrinsic patterns. Then, we examined the influence of intrinsic patterns on the adaptation of sensorimotor synchronization according to various temporal constraints. To set the adaptation task, experiment 1 was designed to examine the relationship between tempo and coordination pattern for expert jugglers. Based on experiment 1, juggling in accordance with the tempo change was performed as adaption task in experiment 2, and we compared the performances of the jugglers from the viewpoint of the intrinsic pattern. In experiment 1, participants performed juggling by adjusting catch timing to beep timing in ten conditions with the interval from 260 to 620 ms in steps of 40 ms. Results of experiment 1 presented that when the juggling tempo is fast, the coordination pattern with “rhythmic” frequency characteristics appeared. By contrast, when the tempo is slow, the coordination pattern with “discrete” frequency characteristics appeared. That is, jugglers should switch their coordination patterns when performing under various tempo conditions. In experiment 2, we compared the adaptability to perform juggling under temporal constraints among intermediate jugglers who have different intrinsic coordination patterns acquired through a previous learning process. The adaptation task required participants to adjust their catch timing to a gradually changing tempo. Participants performed juggling under two conditions: gradually ascending and descending tempo ranging from 300 to 600 ms. The results of experiment. 2 showed that participants who had a discrete pattern showed a significantly better adaptation than participants who had a rhythmic pattern. Furthermore, this result of adaptation was not related to juggling experience. This suggests that an intrinsic pattern characterized by different frequency characteristics has the different adaptability to sensorimotor synchronization tasks. Collectively, the degree of adaptability was dependent on the pattern acquired in the early stages of learning.

## Introduction

Motor learning is the process of overcoming intrinsic constraints in order to achieve the task goal (Turvey, 1990). Recent studies of learning of sports or music have shown that overcoming various intrinsic constraints such as physical constraints or environmental constraints is one aspect of learning (Fujii et al., 2010; Miura et al., 2011, 2013). For example, Fujii et al. (2010) showed that expert drummers achieve fast and stable bimanual drumming, overcoming pre-existing tendency of functional asymmetry between left and right hands. Furthermore, the purpose of the motor learning process is not only to achieve a single goal, but also to adapt to various constraints for expertise. That is, for expertise after we acquire fundamental motor skills, we need to adapt to new task constraints and to acquire new motor skills. In this study, we examined this “new learning process after learning of fundamental skills.”

One of the constraints to be adapted for expertise is coordination with external stimuli such as other performers or music. Movement with external stimuli is called sensorimotor synchronization (Repp, 2005; Miura et al., 2011; Repp and Su, 2013). Also, in ball juggling tasks, experts juggle in synchrony with other jugglers or music; thus, sensorimotor synchronization is an important process to increase expertise (Zelic et al., 2012). During juggling with others or music, the timing of throw or catch should be adjusted to the timing of the external stimuli such as a music, and the juggler should change the movement timing or amplitude. Thus, a juggler needs to control the movement of the three balls continuously and in parallel; then, manipulating the event timing to match with the stimuli is difficult.

Moreover, the expertise process is not only limited to stable performance under a specific constraint, but also requires flexible performance under various constraints (Rosalie and Muller, 2012). Skilled swimmers can switch patterns appropriately according to the speed of swimming (Seifert et al., 2013), and skilled cricket players can change their own batting patterns according to the trajectory of the thrown ball (Pinder et al., 2012). Therefore, the expertise process requires not only adjustment to severe spatiotemporal constraints but also adaptation to various constraints by changing their own movement patterns (Komar et al., 2015). Moreover, adaptation to various constraints in the expertise process can be achieved by appropriately switching patterns according to the constraints. Such a switching of patterns is considered to be a critical factor to perform according to the timing constraints, as required in juggling synchronized with music or tempos. Then, in this study, we applied adaptation process to sensorimotor synchronization task with various external sound in individual juggler.

Individual differences in coordination patterns acquired by each learner need to be considered to examine the adaptation process for expertise. In our previous study (Yamamoto et al., 2015), we examined the acquisition process of the coordination patterns in juggling task. This study revealed that there were multiple coordination patterns that acquired by learners, and the unique coordination pattern of each individual was acquired at a very early stage of learning. Moreover, the acquired patterns did not change through the learning process. Some previous studies reported that individual’s movement patterns acquired once are retained even after several years from the learning session (Nourrit-Lucas et al., 2013; Park et al., 2013). That is, movement patterns acquired in a specific set of constraints are preserved as an individual-specific pattern like “habit.”

The individual’s current system architecture (i.e., developmental status) and previous history of activity (i.e., learning and experience) (Thelen et al., 1993) are shaped by continuous interactions among personal, task, and environmental constraints. From this concept, the coordination pattern acquired in the previous process can become a part of the constraint factor as the current intrinsic system in adapting to the new task (Woodworth and Thorndike, 1901; Thelen and Smith, 1994). That is, the intrinsic dynamics, including acquired patterns, might facilitate or inhibit the adaptation process to new constraints. However, when multiple coordination patterns are acquired in the previous process, the adaptability to the new constraints among the patterns is not necessarily same.

Ikegami et al. (2010) reported that participants who learned through a discrete movement pattern in the pre-learning process in continuous leaching task showed an almost complete transition to a new task that needs to be executed with a rhythmic movement pattern and that tasks for the opposite direction showed almost no transition. This study indicated that those with intrinsic movement patterns acquired in the previous learning process have “asymmetric” adaptability to new constraints. This asymmetry of adaptation between rhythmic and discrete movement was reported by some studies (Ben-Tov et al., 2012; Leconte et al., 2016) that explained these asymmetries of adaptability according to differences in motor primitive between rhythmic motion and the discrete motion in continuous motion (Howard et al., 2011; Sternad et al., 2013; Giszter, 2015). That is, discrete movements are not made of truncated rhythmic movements, and rhythmic movements do not consist of concatenated discrete movements (Leconte et al., 2016). In other words, it can be said that the discrete movement is a series of movements by connecting the movements which are originally controlled separately. On the contrary, rhythmic movement is a continuously controlled movement with no subdivision in a series of movements. That is, even a continuous movement that looks the same may be controlled by several different processes.

As mentioned above, in our previous study (Yamamoto et al., 2015), in the learning process of the 3-ball cascade juggling task that is the fundamental skill of juggling, the movement patterns acquired by the learner were differentiated into a few attractors. The acquired movement patterns were roughly divided into two groups of high ratio pattern and low ratio pattern. This difference in patterns was described by collective variable “dwell ratio” which means the ratio of duration of ball loaded in hand for one hand cycle, and this index could express coordination pattern of juggling.

Juggling involves throwing and catching the ball alternately with the left and right hand. Each hand catches the ball thrown by the other hand. There is a duration during which the hand is empty between the throw and the catch, but this duration varies depending on the height of the thrown ball or the position of the catch. This pattern with relatively shorter duration of ball loaded in hand is called as low dwell ratio pattern (i.e., duration of ball unloaded is relatively long). While, a pattern with relatively longer duration of ball loaded in hand is called as high dwell ratio pattern (Beek and Turvey, 1992; Hashizume and Matsuo, 2004). This high ratio pattern also has specific characteristic in hand movement. This high ratio pattern has a stop phase during the period from the ball catch to the ball throw. Thus, the high ratio pattern is suggested to have a characteristic of continuous “discrete” movement patterns. By contrast, in the low ratio pattern, the ball holding time is relatively short. One series of movement from the ball throw to the next ball throw is smooth, and the speed change is small. Thus, the low ratio pattern is suggested to have a characteristic of continuous “rhythmic” movement patterns. Our previous study showed the possibility that the two attractors in juggling were “discrete” and “rhythmic” movement patterns (Yamamoto et al., 2015).

Discrete and rhythmic movements are distinguished according to the movement frequency (Levy-Tzedek et al., 2010, 2011). They indicated that high-frequency movements are highly rhythmic and low-frequency movements are more discrete-like. Then, we investigated the adaptability among coordination patterns in juggling with a focus on the difference in the frequency characteristics through an experimental manipulation of movement frequency. From these studies, it was assumed that different patterns and different strategies are taken in juggling according to various tempos. If the tempo of juggling is fast, the juggler needs to catch and throw the ball smoothly and quickly. Therefore, it is expected to become a “rhythmic” pattern that smoothly connects between events. On the contrary, if the tempo is slow, there will be duration between catch and slow event, so it will be a “discrete” movement that has stop phase of moving between the events.

From both the relationship between tempo and movement frequency and the feature of movement of juggling, it is hypothesized that rhythmic patterns and discrete patterns will appear in response to fast tempo and slow tempo. In addition, from the viewpoint of asymmetry of adaptation among motor primitives, it is hypothesized that participants with discrete patterns intrinsically will exhibit better adaptability.

The purpose of this study is to investigate the effect of individual differences in a learner’s intrinsic movement patterns on adaptability to new constraints. In this study, we applied adaptation process to sensorimotor synchronization task with external sound in individuals. Then, we examined the adaptability for intermediate jugglers who have already learned fundamental skills (i.e., three-ball juggling) in juggling. Before that, to set the adaptation task, first as experiment 1, we examined a change of movement patterns, focusing on frequency of the juggling tempo for expert jugglers. Based on the results of experiment 1, we examined the effect of individual differences in coordination patterns among learners on the performance of adaptation to various temporal constraints for intermediate jugglers as experiment 2.

## Materials and Methods

### Participants and Protocol

Seven expert jugglers (males; mean age: 19.5 ± 0.5 years) who can perform -5 or 7-ball juggling participated in experiment 1. The definition of the expert juggler was based on the criteria given by Beek (1989). We investigated the emergence of coordination patterns, focusing on the frequency characteristics in accordance with the change in temporal constraints. Participants were asked to perform 3-ball juggling under ten conditions with the metronome beep intervals of 260, 300, 340, 380, 420, 460, 500, 540, 580, and 620 ms. These 10 steps of tempo were decided to be recognizable width as the different tempo within the fastest and slowest feasible range by performing preliminary experiments. Each tempo condition consists of 65 beeps. Participants were only required to perform 3-ball cascade juggling with adjusting the catch timing to the metronome beep timing (created by Audacity version 2.1.2.0^1^). We did not instruct them about the coordination pattern or height of throwing a ball in each condition. Participants were asked to adjust to catch timing as soon as possible after the beep began. They performed in ascending order from fast tempo condition (260 ms) and descending order from slow tempo condition (620 ms). The ascending and descending orders were counterbalanced among participants.

Meanwhile, 11 intermediate jugglers (8 males and 3 females, age 20.6 ± 2.7 years) who can only perform 3-ball juggling participated in experiment 2. All the participants in Experiments 1 and 2 did not have any specific experience related to sensorimotor synchronization, such as dance and music except juggling. We set an adaptation task with two conditions to juggle in accordance with the beep sound whose tempo gradually changes. In the Up condition, the interval of the metronome sound gradually ascends from 600 to 300 ms in increments of 3 ms. Meanwhile, in the Down condition, the interval descends in steps of 3 ms from 300 to 600 ms. One trial consisted of 101 beeps. As with experiment 1, this tempo width was also determined based on preliminary experiments. In the preliminary experiment, the intermediate juggler seemed to be unstable when perform juggling with sound and was observed to have more difficulty to juggle under very fast and very slow conditions. Therefore, the upper and lower limits of tempo stimulation used in experiment 2 were narrowed. Moreover, to verify the flexible switching of patterns, we changed the tempo within the trial, and then the tempo was gradually changed because abrupt changes in the tempo might be difficult for intermediate jugglers. Furthermore, to verify the intrinsic pattern of each participant, participants performed juggling with free tempo without metronome sound for 30 s as the Preferred condition. Same as in experiment 1, participants performed 3-ball juggling in accordance with sound. Each condition was performed in three trials, and the order of the Up and Down conditions was counterbalanced among the participants. The study was approved by the Ethics Committee of the Graduate School of Arts and Sciences, The University of Tokyo, and all participants gave their written informed consent to participate.

### Data Collection

An optical motion capture system with four cameras (100 Hz, Optitrack, Natural Points) was used to record the participant’s movements during all trials. Three balls (6.6 cm in diameter and 130 g in weight) were covered with reflective tape. The cameras were placed around the participant so that the participant and the balls being juggled were all in view. The three-dimensional coordinates of the markers (x-axis: anterior-posterior, y-axis: vertical, z-axis: lateral-medial) were calculated using Motive software. Reconstruction of the known marker positions on the calibration frame before each learning session yielded residual errors of reconstruction of less than 1 mm for each coordinate. Moreover, the metronome sound generated by the PC through experiments 1 and 2 were recorded via a data acquisition device (1000 Hz, NI-USB 6218, National Instrument) and recorded using Labview (National Instrument). The motion data and metronome sound were synchronized by outputting a synchronization signal from Optitrack and recording it on Labview at the same time as the sound data.

### Data Reduction

The obtained metronome sound data and the synchronization signal (1000 Hz) were down-sampled to 100 Hz using the thinning method to synchronize with the motion data of Optitrack. We synchronized the metronome sound data with the Optitrack data using the down-sampled synchronization signal. Digitized coordinates of the three balls were identified and tracked using the Motive. Marker switching or misidentification of two adjacent markers during automatic tracking was corrected manually. Missing data points due to a brief occlusion were interpolated automatically via the spline method using the Motive. Following the widely accepted technique of Winter (2005), a high-frequency noise was reduced using a zero-phase (bidirectional) digital filtering with second-order Butterworth filter for the digitized data. The cutoff frequency was determined using residual analysis. The filtered displacement values along the y-axis were differentiated to obtain the velocity of the ball and hand movement in the vertical direction. The velocity profile of the ball was used to identify the moments as throws, catches. The timing of the throw was defined as the time that the ball velocity reached its positive peak, and the timing of the catch was defined as the time that the ball velocity reached its negative peak. Therefore, the velocity profile of the hand was used to describe the movement pattern during juggling.

### Data Analysis

In Experiment 1, Coordination Pattern Index and Dwell Ratio were calculated as indices of coordination patterns. The calculation method was described below. The movement pattern performed corresponding to the tempo was described with a focus on the frequency characteristic. As mentioned in the Introduction section, in the additional analysis of my previous research (Yamamoto et al., 2015), the difference between the multiple low- and high-ratio patterns could be described by the velocity data of the hand movement. Thus, the velocity data in the vertical direction of the wrist marker were used for describing the coordination pattern. The first six catches (i.e., 1 cycle in 3-ball juggling) out of the 65 times were excluded from the analysis range because the juggling movement becomes unstable right after a trial starts.

**FIGURE 1:**
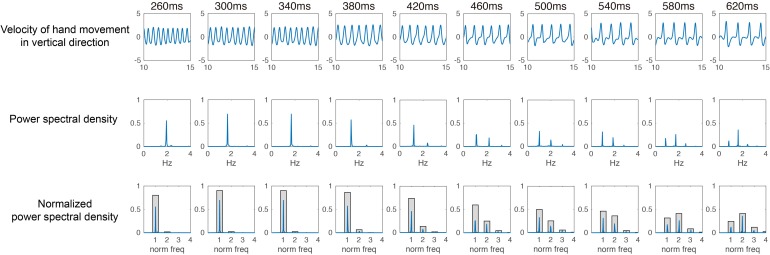
Typical example of movement patterns among different tempo conditions for one expert juggler.

The frequency characteristics were analyzed by calculating the power spectral density after Fourier transformation for the velocity data of vertical hand movement (cf. Yang et al., 2014). From the calculated spectrum density, we divided the whole frequency components into fundamental frequency components and 2, 3, and 4 times frequency components. The proportion of each frequency component was calculated within a range of ± 10%, and the proportion of the fundamental frequency was defined as the Coordination Pattern Index (see **Figure 1**).

Furthermore, we investigated the relationship between Coordination Pattern Index described by frequency characteristics and Dwell Ratio used in previous research (Yamamoto et al., 2015). The Dwell Ratio was calculated by the ratio of the time loaded from catching the ball to throwing it in one cycle (hand cycle time) from the throw of each hand to the next slow.

In experiment 2, to investigate whether the participants can adapt to the task, we calculated %Asynchrony which is the absolute error between each catch and each beep timing. This %Asynchrony means the accuracy of performance of juggling in accordance with changing metronome beeps. We removed the first 6 out of the 101 catches from the analysis range, then analyzed 95 catches. In three participants, the number of catches included in one trial was small (at least 63 catches) because ball falling was seen during the trial. Furthermore, because the tempo gradually changes during one trial, the ratio of absolute error for the requested tempo is calculated as the % Asynchrony. In addition, to describe the intrinsic pattern of each learner, the frequency characteristics of the vertical velocity of the hand movement was analyzed, and the proportion of the fundamental frequency component in all frequency components was defined as the coordination pattern index. Furthermore, the frequency characteristics of the pattern concerning the transition of the movement pattern during the adaptation task of 8 out of 11 participants (the data of the 3 participants were missing) were examined.

We divided the analysis range 96 times into left and right hands (each 48 cycles) and calculated the change in the fundamental frequency ratio (Coordination Pattern Index) in steps of three cycles (6% of the one trial). As the Flexibility of Pattern Transition Index, the standard deviation in the trial of this pattern transition index was calculated. We examined whether the adaptive performance index can be explained by an individual’s intrinsic pattern. Furthermore, we investigated whether the adaptive performance index can be explained by the flexibility of an individual pattern transition.

### Statistical Analysis

Pearson’s correlation analysis was performed for statistical analysis in both experiments 1 and 2. In experiment 1, two trials of each tempo condition of seven participants were used for analysis. Ten tempo conditions were considered as independent variables, and the fundamental frequency component proportion of the spectral density of movement pattern under each condition was considered as a dependent variable. In experiment 2, the performance of the adaptation task was considered as the dependent variable, and the coordination pattern index that indicates individual intrinsic pattern was considered as the independent variable.

Moreover, the correlation analysis using the participant’s juggling experience (practice period described by the month unit) as independent variables was conducted to examine the determinants of adaptation performance. We also examined the relationship between the flexibility of pattern transition index and the adaptive performance. The level of significance was set to 5% in each case.

## Results

### Multiple Patterns Depending on Frequency of Juggling (Exp. 1)

The purpose of experiment 1 was to clarify the change of the coordination pattern appearing in accordance with the temporal constraint by describing the frequency characteristics of the hand velocity pattern. **Figure 1** shows the typical examples of the speed (upper row), power spectral density (middle), and normalized power spectral density (lower row) of one expert juggler. Around the fast tempo of 260 ms, the hand velocity pattern showed a smooth sinusoidal waveform with less change of velocity. As the tempo slowed down, the velocity pattern showed a stop phase or a fast frequency component between catch (negative peak of velocity) and ball throw (positive peak of velocity).

In the 620 ms condition, which is the slowest tempo, the fast frequency component significantly appeared in the phase between the catch and the ball throw. Regarding velocity properties, the frequency characteristics of the coordination pattern were different depending on the tempo. Under the 260 ms condition, the cycle time of each hand was doubled to 520 ms (1.92 Hz). A peak of spectrum density appeared around 1.92 Hz. Moreover, under the 620 ms condition, the slowest tempo, the peak of fundamental frequency components appeared around 0.81 Hz corresponding to a hand cycle time of 1240 ms.

Furthermore, peaks also appeared around 1.61 Hz, which is the doubled frequency component, and around 2.43 Hz, which is the tripled frequency component. Interestingly, the peak of the frequency spectrum characteristic accompanying the descending tempo appeared at an integral ratio of the fundamental frequency. This suggests that the juggling task is temporally constrained tightly.

**FIGURE 2:**
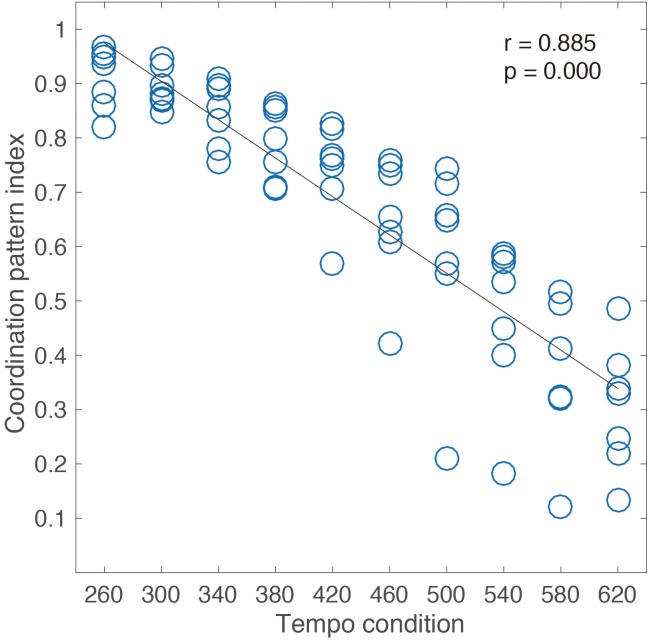
Correlation analysis between ten tempo conditions and coordination pattern index of each condition for seven expert jugglers.

**Figure 2** shows the strong correlation (Pearson’s *r* = 0.88, *p* < 0.05) between tempo conditions and coordination pattern index for the seven expert jugglers. This result shows that the rate of the fundamental frequency decreases from the fast tempo to the slow tempo. Hence, the frequency characteristics of the movement pattern were found to change according to the juggling tempo. The movement pattern composed only of fundamental frequency appearing under such a fast tempo condition is a “rhythmic” movement pattern having a sine curve-like property of velocity.

Meanwhile, the movement pattern including double frequency component appearing under the slow tempo condition was a “discrete” movement pattern. We also examined the relationship between the frequency characteristic index of coordination patterns and the “dwell-ratio” index of the coordination pattern (Yamamoto et al., 2015). We performed a correlation analysis between the coordination pattern index described by fundamental frequency ratio and dwell ratio that shows the unique pattern of 18 participants, including seven expert jugglers in experiment 1 and 11 intermediate jugglers in experiment 2.

**FIGURE 3:**
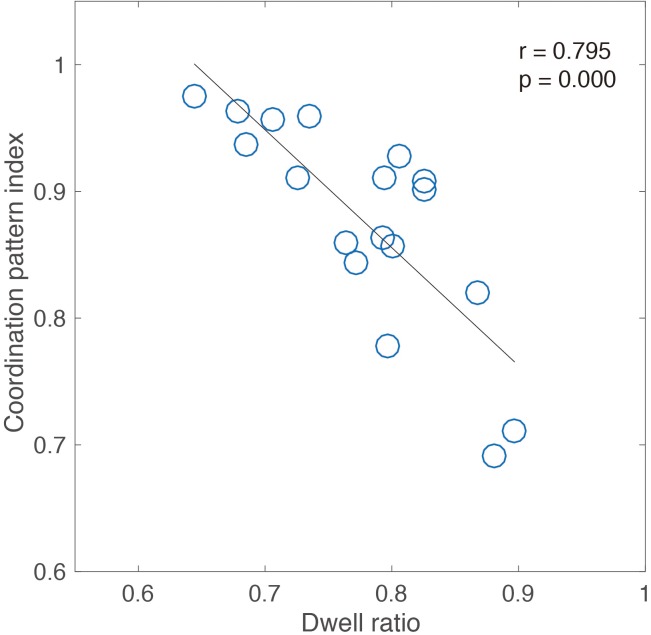
Correlation analysis between coordination pattern index and dwell ratio for seven expert jugglers and 11 intermediate jugglers.

**Figure 3** shows the significant correlation found between the coordination pattern index and dwell ratio (Pearson’s *r* = 0.79, *p* < 0.05). It shows that the high ratio pattern was a “discrete” pattern, and the low ratio pattern was a “rhythmic” pattern. These results of frequency characteristics for expert jugglers means that an appropriate pattern appears corresponding to the temporal constraint in juggling. That is, to accurately perform sensorimotor synchronization with auditory stimulus, it is important to maintain a rhythmic pattern with a faster tempo and a discrete pattern with a slower tempo. Moreover, it was necessary to switch flexibly among these patterns.

### Asymmetric Adaptability Between Preferred Patterns (Exp. 2)

**FIGURE 4:**
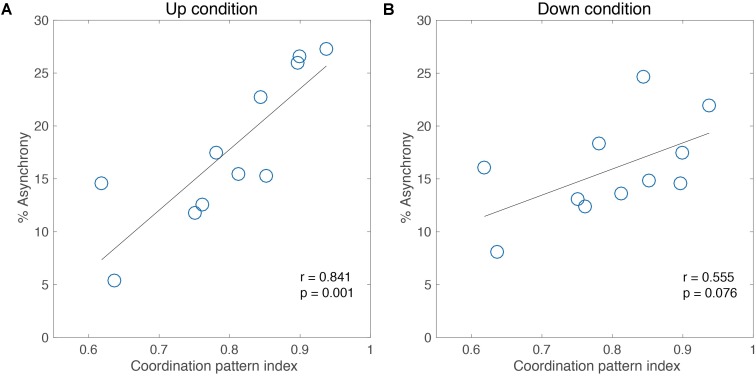
Correlation analysis between performance of adaptation task and coordination pattern index for 11 intermediate jugglers in the Up condition **(A)** and the Down condition **(B)**.

Next, for experiment 2, we examined the influence of the individual intrinsic pattern of the participants on adaptability for intermediate-level jugglers. **Figure 4** shows the correlation between the intrinsic coordination pattern index of each participant and the accuracy performance (% Asynchrony) of the adaptation task in each of the tempo Up and Down conditions. Pearson’s correlation analysis showed a significant correlation between the coordination pattern index and the % Asynchrony for the Up condition (Pearson’s *r* = 0.84, *p* < 0.01, **Figure 4A**).

**FIGURE 5:**
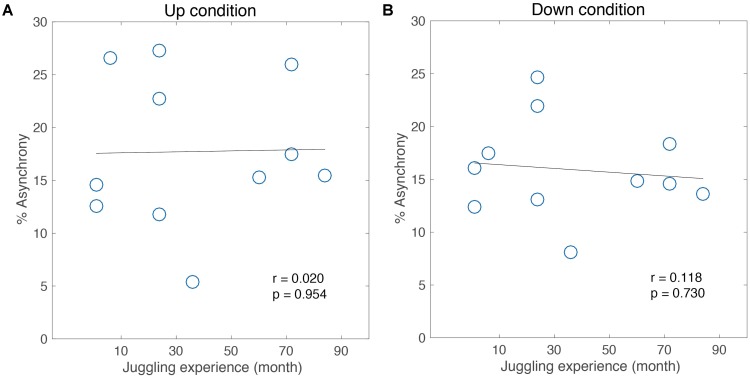
Correlation analysis between performance of adaptation task and skill level for 1 intermediate jugglers in the Up condition **(A)** and the Down condition **(B)**.

Also, a moderate, marginally significant correlation was found in the Down condition (Pearson’s *r* = 0.55, *p* = 0.07, **Figure 4B**). Thus, the adaptability in the performance was different under temporal constraints among the intrinsic movement pattern of each individual. Meanwhile, no significant correlation was found in the skill level (Pearson’s *r* = 0.01, n.s. for the Up condition, **Figure 5A**; Pearson’s *r* = 0.11, n.s. for the Down condition, **Figure 5B**). That is, this difference of adaptability was not depends on the experience of juggling but is relate to an intrinsic movement pattern.

**FIGURE 6:**
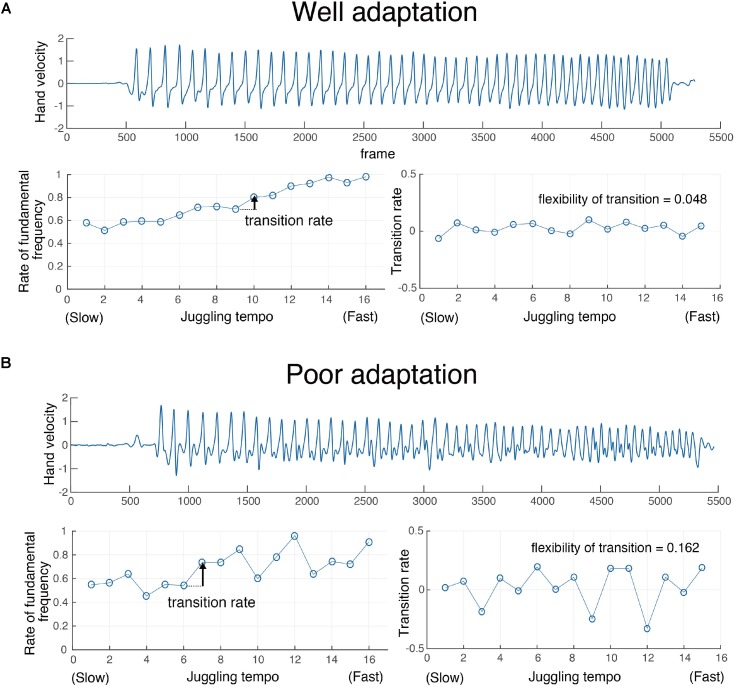
Typical examples of pattern transition for one well-adapted participant **(A)** and one poorly adapted participant **(B)**.

Moreover, we examined the transition of coordination patterns in accordance with tempo changing during the adaptation task. The flexibility of the pattern transition index was calculated as the rate of change of the ratio of the fundamental frequency in each of the three cycles, and correlation analysis with the adaptive performance was performed. **Figure 6** shows a typical example of a transition of coordination pattern in the Up condition (**Figure 6A**) of one participant who showed relatively good adaptation and one participant who showed relatively poor adaptive performance (**Figure 6B**).

**FIGURE 7:**
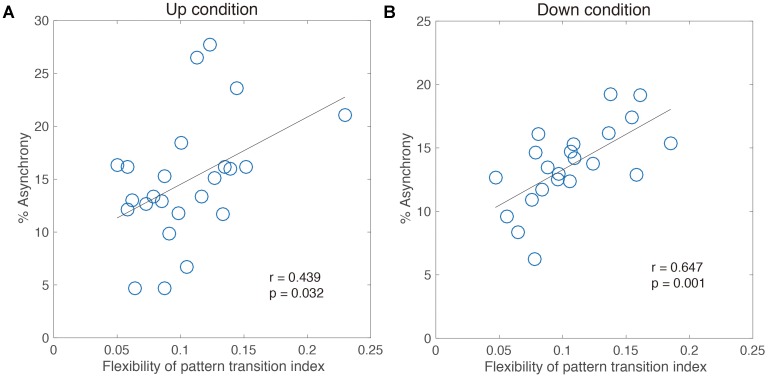
Correlation analysis between performance of adaptation task and flexibility of pattern transition index for 11 intermediate jugglers in the Up condition **(A)** and the Down condition **(B)**.

A significant correlation (Pearson’s *r* = 0.44, *p* < 0.05 for the Up condition, **Figure 7A**; Pearson’s *r* = 0.64, *p* < 0.05 for the Down condition, **Figure 7B**) between % Asynchrony and flexibility of pattern transition index was noted. These results indicated that the flexible transition in accordance with the tempo is related to better adaptive performance. That is, the difference in adaptation performance among an individual’s intrinsic movement pattern depends on the possibility of flexible pattern switching.

## Discussion

In this study, we examined the adaptability of multiple-degrees of freedom tasks by focusing on the diversity of intrinsic patterns acquired in the learning process of fundamental skills in juggling. Through a frequency analysis of the expert jugglers, experiment 1 revealed the diversity of coordination patterns in accordance with juggling tempo. This result also suggests that adaptation to a wide range of temporal constraints may be achieved by appropriate switching of coordination patterns. Therefore, in experiment 2, we examined the adaptability to the temporal constraints among individual intrinsic patterns. Participants who have discrete patterns showed higher adaptability than participants who have rhythmic patterns. In addition, these better adaptations were related to the flexible pattern switching in accordance with changing tempo stimulus. In other words, when adapting to sensorimotor synchronization with wide-width constraints in juggling, it is important to change the coordination patterns according to the constraints.

### Diversity of Movement Patterns in Juggling

Yamamoto et al. (2015) reported that the coordination patterns acquired by learners in juggling are differentiated into the low ratio pattern and high ratio pattern that correspond to the “rhythmic” movement pattern and the “discrete” movement pattern, respectively, as shown in experiment 1. In the low ratio pattern, the juggler throws a ball in series of flow after catching it. Therefore, the hand movement becomes a smooth “rhythmic” pattern. Conversely, the high ratio pattern is a pattern in which the hand is stopped once after the ball catch. Thus, the high ratio pattern corresponds to the “discrete” movement pattern.

These results are consistent with the change in dwell ratio according to the juggling tempo reported by Beek and Turvey (1992). Based on these reports and the significant correlation between the fundamental frequency ratio and the dwell ratio in this study, the multiple coordination patterns differentiated in the early stage of learning in the report of Yamamoto et al. (2015) each have different frequency characteristics.

The results of the current study and those of previous studies suggest that the diversity of movement patterns occurring in juggling may be due to differences in motor primitives used for control (Schaal et al., 2004; Hogan and Sternad, 2007, 2012, 2013; Hira et al., 2015). Although whether the learner intends to control the frequency characteristics during the learning process is unknown, control based on different movement primitives might be performed as a result due to difference in strategy. This suggests that control by primitives with different frequency characteristics may cause diversity in the motor task; as such, temporal components such as tempo or rhythm are the key to learning. Furthermore, in juggling, control of slow and catch events is added to continuous movement. In the adaptation process to sensorimotor synchronization with auditory stimuli, the control strategies of these events become to be an important factor determining the adaptability.

### Subdivision Benefit to Adaptation in Continuous Movement

Experiment 2 showed that the differences in adaptability were not related to the skill level. In this task, the catch or throw events must be intentionally controlled during cyclic juggling movement. Juggling with a discrete pattern is separated once by a cyclic movement in a ball catch event. Therefore, changing the movement to match the timing of the catch with the beep of the metronome is possible in cyclic juggling compared to rhythmic juggling. That is, discrete patterns may possibly be the affinity to adaptation tasks. The subdivision of movements may be the key point when adapting to external inputs, such as adapting movements to sounds or matching the movements of other jugglers.

van Santvoord and Beek (1996) reported that jugglers fixed their throwing position as an anchor to improve the stability of performance. This means that providing an anchor in a specific phase stabilizes the movement and adjust/correct the motion. Moreover, in tapping motion synchronized with sound, tapping performance is more stable to synchronize when the sound and tap is subdivided into 1: 2 and 1: 3 rather than into 1: 1 (Repp, 2003). This is called “subdivision benefit” and it plays an important role in supporting temporal accuracy in sensorimotor synchronization with auditory stimuli (Goebl and Palmer, 2009; Repp, 2010). That is, in a periodic movement, taking a strategy such as taking a beat somewhere in a series of movements and subdividing the movement facilitates control and enhances the accuracy of sensorimotor synchronization. In juggling, it is possible to take a beat by subdividing a series of movements by catch and throw events. However, the rhythmic pattern has the characteristic of performing juggling in a series of movements; thus, it has been difficult to obtain the subdivision benefit as compared with the discrete pattern that has a stop phase and subdividing a series of movements while performing juggling. As such, the adaptability between the two patterns was different. In the adaptation process, individual movements must be reconstructed according to the input of the new environment to be adapted (Kostrubiec et al., 2012). Therefore, it is suggested that, instead of controlling periodic movement as a series of movements, subdividing and controlling specific elements as anchor points facilitates control and helps to correct and reconstruct their own movements. Thus, the difference in the strategy may determine the degree of learning and adaptation. Furthermore, consideration from the perspective of attractor dynamics regarding the difference in adaptability of patterns obtained in the fundamental learning process can be considered.

### Difference in Adaptability From the Perspective of Attractor Dynamics

Another reason for the difference in adaptability might be caused by the difference in attractor layouts between discrete and rhythmic movement patterns. In experiment 2 of this study, the metronome was changed from a fast tempo to a slow tempo and vice versa. To accurately perform sensorimotor synchronization with this sound stimulus, as shown in experiment 1, it is important to perform according to a rhythmic pattern in a fast tempo band and a discrete pattern in a slow tempo band. Moreover, it was necessary to switch flexibly among these patterns. For this adaptation task, participants who intrinsically had discrete patterns showed flexible switching ability between discrete patterns and rhythmic patterns. By contrast, participants who intrinsically had rhythmic patterns were not able to do flexible switching sufficiently. This suggests that participants who had acquired discrete patterns in the previous learning process acquired rhythmic movements as local attractors.

Some studies have recently reported asymmetric adaptability between rhythmic and discrete movement (Ikegami et al., 2010; Leconte et al., 2016). According to Schaal et al. (2004), the primitive of the discrete movement pattern includes the primitive of the rhythmic movement pattern. These two motor primitives are not totally independent and contain part of the rhythmic motor primitives in the sequence of discrete motor primitives. That is, there may be rhythmic characteristics on the extension line of an attractor with discrete characteristics, but an inverse phenomenon may not be observed.

As such, learners who have discrete patterns may show better adaptive performance.

Meanwhile, the restraint of deviation from intrinsic movement pattern is considered as the other possibility of factors determining the difference in the adaptability. Movement patterns acquired in the learning process of fundamental skills of juggling are self-organized into stable attractors through the frequency locking of temporal variables constituting juggling (Yamamoto et al., 2015). This attraction to stable pattern supports the stable performance of 3-ball juggling. However, this study requires a deviation from an intrinsic pattern because adapting only by using their own intrinsic patterns was difficult. Therefore, entrains into the acquired intrinsic pattern preventing the transition to requiring deviation is possible. Considering this point, the difference of adaptability is caused by difference in the possibility of the deviation from the intrinsic patterns. Thus, regarding the asymmetry of the adaptability as seen in this research, it may be attributed to various factors. Further examination is necessary for identifying critical factors. Then, to consider individual differences in learning and adaptation from various viewpoints could be important.

### Individual Differences in Learning and Adaptation

The transfer or adaptation has been investigated by focusing on the similarity of tasks that are likely to transfer (Hazeltine et al., 2007) and the kind of movement components that are transferred (Snapp-Childs et al., 2015). Less attention has been given to the diversity of the movement patterns acquired by each individual learner. Furthermore, in studies on individual differences in learning, individual factors such as general cognitive ability and searchability (Ackerman, 1987) have been focused on, while individual differences in task-dependent movement patterns have been rarely considered.

However, in recent years, the asymmetry of adaptability (e.g., Ikegami et al., 2010) among multiple movement patterns with different motor primitives has been reported in reaching tasks. This indicates the importance of examining the diversity of motor learning processes. The motor learning process can be regarded as a phenomenon of differentiation into multiple of paths, which is not limited to a single path derived from the interaction between constraints.

From this perspective, learners who follow different paths have different adaptability to follow paths for new adaptation task. As revealed in experiment 2, in intermediate jugglers, individual differences in intrinsic patterns acquired during the learning process of fundamental skills of juggling cause the individual differences in adaptability. This strongly suggests that the diversity of individual patterns and individual differences in multi-degree of freedom systems should be considered. In other words, it means that it is necessary to consider that there are various paths for learning. This concept has been accepted in a variety of study fields.

### Long-Term Learning Process Based on Differentiation Phenomena

Integrating the results of this study with those of Yamamoto et al. (2015), we found that learners adapted to new constraints while retaining intrinsically different patterns of rhythm and discrete in the fundamental learning process of juggling. As such, different adaptation processes occurred between those patterns. These learning and adaptation processes can be interpreted as a differentiation phenomenon that follows various paths through a long process. This concept is used in infant motor development and understanding the evolution process in biology, and it is considered that common understanding with motor learning is possible. Several developmental studies, such as that by Thelen et al. (1987), described various movement patterns for infants as a process of bifurcation from a basic pattern resulting to various patterns. Meanwhile, in biology, the evolution of a species (pattern) is regarded as a phenomenon in which organisms originally of the same species systematically differentiate into species with systematically different characteristics, and this concept is called cladogenesis (Futuyma, 2010). In other words, the time development of evolution is the process by which the species differentiate into those with different properties. Likewise, the learning process can possibly be regarded as a process of differentiating into patterns having various characteristics with the development of time. This time-developing phenomenon with differentiation has been described via an “epigenetic landscape model” (Waddington, 1957). In this model, differentiation phenomena follow the rule of transition between states to adapt to the environment over time and may be related to the process of motor learning over a long period. Based on the results of this research with Yamamoto et al. (2015), the learning of juggling is differentiated into different patterns of rhythm and discrete movement, then it adapts to new constraints and changes to a different pattern.

Furthermore, stable pattern acquired in the learning process is considered to be among the “habits of movement” (Barrett, 2014). This individual stable movement pattern is expected to be acquired and retained by the fundamental skill of a specific motor task (Park et al., 2013). Interestingly, in experiment 2, participants with 6 or 7 years of juggling experience exhibited adaptive performance comparable to that of participants for several months.

This suggests that the juggling practice with a specific movement pattern made the movement pattern of the juggler stronger, suggesting the possibility that deviations were hindered. In this case, the learner might have to reconstruct the pattern tracing backward. Considering the reversibility going back along the followed route leads to understanding of the movement learning process.

## Conclusion

In conclusion, this study showed that the intrinsic movement patterns of learners influence the adaptability to new constraints. In particular, flexibly switching patterns according to the constraints was important in sensorimotor synchronization with a wide external auditory stimulus. However, the results indicated that the difference in the intrinsic patterns acquired through the fundamental learning process determines the adaptability to the sensorimotor adaptation task. This means that the individual habit of movement formed in the early stages of learning plays an important role in the long-term learning process.

## Author Contributions

KY, MS, and KK contributed to the conception and design of the work, the data analysis, and interpretation of data. KY contributed to the data acquisition and wrote the manuscript. MS and KK commented on and revised the manuscript.

## Conflict of Interest Statement

The authors declare that the research was conducted in the absence of any commercial or financial relationships that could be construed as a potential conflict of interest.

## Footnotes

1 http://audacity.sourceforge.net/
